# 开胸术后疼痛的防治进展

**DOI:** 10.3779/j.issn.1009-3419.2012.08.07

**Published:** 2012-08-20

**Authors:** 夏轶 闾, 湘 徐, 艾则麦提·如斯旦木, 永清 王

**Affiliations:** 310003 杭州，浙江大学附属第一医院胸外科 Department of Thoracic Surgery, the First Hospital Affiliated to Zhejiang University, Hangzhou 310003, China

**Keywords:** 开胸术后疼痛, 关胸缝合技术, 肋间神经, Post-thoracotomy pain, Chest closure technique, Intercostal nerve

## Abstract

本文对开胸术后疼痛的预防和治疗策略作一简明综述，并主要关注外科手术操作技术的改进对于减轻开胸术后疼痛的作用，同时也兼顾了一些麻醉药物方面的进展。尽管已有诸多相关研究在手术方式和药物镇痛两个方面对治疗和预防开胸术后疼痛进行了一些探索，但由于疼痛的个体差异、手术者操作技术的差别以及研究样本量的限制，目前尚难得出确切的结论支持其中的某种方法为“最优”选择。这一领域仍期待着临床专家的深入探索和研究。

## Abstract (en)

This article provides a concise review of post-thoracotomy pain-relief strategies, focuses on surgical factors that have been investigated to reduce post-thoracotomy pain, and explores the effectiveness of various treatments for this condition. Although some interventions (both procedural and pharmacologic) have been investigated in both preventing and treating post-thoracotomy pain syndrome, definitive studies are lacking and firm conclusions cannot be drawn. This review elucidates mechanisms and some surgical techniques which can relieve post-thoracotomy pain.

开胸术后急、慢性疼痛的发生对患者术后呼吸功能、日常生活的恢复均造成很大影响，甚至导致一些严重的并发症。因此寻找一种有效的方法，包括改良手术方式、改进缝合方法、术后合理止痛，都值得研究和探讨。本文综述相关文献，以提供临床应用参考。

## 1 手术方式的影响和改进

根据胸部解剖的特点正确选择手术切口开胸是手术成功的重要环节。常用的胸部切口有后外侧切口、前外侧切口、胸骨正中切口、胸腹联合切口和侧胸小切口。在开胸手术过程中，肋间神经很容易受到损伤，是造成术后急、慢性疼痛的重要原因之一。其它如肋间肌肉损伤、肋骨骨折、过分牵开肋骨或胸骨造成骨质破坏、胸壁肌肉缝合错位、胸管放置等也是引起术后疼痛的一系列因素^[1]^。很多研究^[2, 3]^认为肋间神经损伤和肌肉损伤是术后疼痛发生的先决条件。因此，如何在术中避免肋间神经的损伤并减少肌肉损伤成为很多学者研究的课题，一些改良的手术方式也相继产生。

### 1.1 切口选择

最近的一项研究^[4]^统计了184例接受开胸手术的患者，其中141例行后外侧切口，43例行腋前线切口，结果显示接受后外侧切口手术的患者比接受腋前线切口手术的患者在1个月、2个月、4个月、6个月、9个月、12个月的疼痛评估中承受更重的疼痛。由此认为在选择切口时应兼顾患者术后疼痛的困惑。王永清等^[5, 6]^报道保留背阔肌的开胸术在胸腔内手术中的应用经验，认为与后外侧开胸手术比较，腋前线切口术后疼痛轻，上肢运动影响小。

### 1.2 肋间肌瓣保护

在开胸手术中需要用到胸廓撑开器。在撑开器牵开切口过程中会压迫肋间肌肉和肋间神经导致其损伤，引起术后疼痛。因此，如何避免胸廓撑开器对肋间神经的损伤是值得关注的问题。Cerfolio等^[7]^在一项预期试验中，将114例开胸手术的患者随机分成两组：实验组（分离肋间肌瓣）和对照组（不分离肋间肌瓣），由同一个外科医生进行手术，手术切口为后外侧切口，所有患者术后均应用相同药物及剂量进行术后疼痛的干预。实验组中，小心地剥除第5肋下的肋间肌肉形成游离的肋间肌瓣，以避免胸廓撑开器压迫肋间肌肉及神经，而对照组则没有游离肋间肌瓣，胸廓撑开器直接压迫肋间肌肉及神经。术后用数字评定量表、视觉疼痛评分、口头描述量表评估患者1周、2周、3周、4周、8周、12周的疼痛情况并记录术后镇痛药的使用量。结果表明：实验组术后短期及长期的疼痛均有明显降低（*P*=0.004）。在另一项回顾性实验中，Sakakura等^[4]^得出游离肋间肌瓣组比非游离肋间肌瓣组在术后第1个月内的疼痛较轻，但是长期的疼痛无明显差异。因此，肋间肌瓣游离技术在开胸术中的应用对预防开胸术后早期疼痛的作用是肯定的，但对后期慢性疼痛的作用还有待于进一步研究与验证。

### 1.3 关胸缝合技术

肋间神经的损伤不仅仅在开胸过程中，术中关胸缝合时也会因缝合技术压迫肋间肌肉和肋间神经，从而引起长期的术后疼痛。因此避免神经压迫的关胸缝合技术逐渐成为热门的研究课题。在Cerfolio等^[8]^研究中，把接受开胸术的280例患者分成两组，一组采用肋间肌瓣内关胸（以第5肋间切口为例，在第6肋骨上钻孔，穿过缝线，紧贴第5肋上缘出针），另一组采用肋间肌瓣外关胸（以第5肋间切口为例，跨肋间，紧沿第7肋骨上缘进针、第5肋骨上缘出针），两组样本各有140例。研究发现肋间肌瓣外关胸组患者的疼痛更多地表现为烧灼样、针刺样疼痛（*P* < 0.003），这正是神经损伤所致疼痛的特点。肋间肌瓣内关胸组所经历的术后疼痛在术后2周、1个月、2个月、3个月均明显较轻，具有明显差异（分别为*P*=0.004、*P*=0.000, 1、*P* < 0.000, 1以及*P* < 0.000, 1）。Sakakura等^[4]^回顾了184例接受腋中线切口开胸术和后外侧切口开胸术的患者，其中87例接受传统关胸方法（以第5肋间切口为例，跨肋间，紧沿第7肋骨上缘进针、第5肋骨上缘出针），97例接受肋间肌瓣edge关胸方法（以第5肋间切口为例，跨肋间，紧沿第6肋骨下缘，在肋间血管和肋骨之间进针、第5肋骨上缘出针），研究发现肋间肌瓣edge关胸手术患者的疼痛评分明显比传统关胸方法的患者低。另一项研究^[9]^将60例接受分离肋间肌肉的后外侧小切口开胸术的患者，一半采用肋间肌瓣内关胸，另一半采用一种改进的肋间肌瓣内关胸方法（以第5肋间切口为例，在第6肋骨上钻孔进针、第5肋骨上钻孔出针），通过3个疼痛量表（analogical-visual, observer verbal order scale and Ramsay’s）对术后48 h的疼痛进行评估，结果除了拉姆塞量表外，另外两个疼痛量表均提示第二组患者疼痛明显减轻。上述几项研究中，研究人员利用不同的关胸方法减轻对肋间肌肉及神经组织的压迫，与传统方法相比，在短期内均有明显的减轻术后疼痛的效果，但是对于术后长期疼痛预防的证据均较缺乏。

### 1.4 电视胸腔镜技术

目前，电视胸腔镜技术已广泛地应用于胸科手术。Miyazaki等^[10]^在一项临床试验中比较了胸腔镜手术、胸腔镜辅助小切口手术和传统开胸术，在患者术前和术后1周、2周、4周、12周、24周，用数字化疼痛量表评估术后疼痛，使用2, 000 Hz（Ab神经纤维）、250 Hz（Ad神经纤维）以及5 Hz（C神经纤维）的电流刺激评估患者切口周围皮肤的电感知阈。结果表明胸腔镜手术组术前与术后的皮肤电感知阈没有变化，疼痛在术后12周之内均消失；而另外两组的皮肤电感知阈明显升高，且12周后的疼痛发生率接近70%。该结果显示了胸腔镜手术对术后疼痛的预防有明显效果。

## 2 术后合理止痛

尽管临床研究对开胸和关胸技术已作了一些改进，但创伤引起的肋间神经和肌肉损伤有时很难避免，并且放置胸腔引流管也会引起胸痛。因此术后麻醉止痛也起着不可缺少的作用。术后麻醉止痛方法有硬脑膜外阻滞、椎旁阻滞、肋间神经阻滞、药物镇痛以及各种方法的联合应用。

### 2.1 镇痛药物

系统服用阿片类药物是开胸术后镇痛的最简单也是最常用的方法，但它会引起很多不良反应，如呼吸抑制、镇静过度、恶心、呕吐等^[11]^。解热镇痛药、非甾体类抗炎药等的联合应用对减轻术后疼痛也有一定的作用^[12]^。

### 2.2 硬膜外阻滞

硬膜外阻滞是开胸术后控制疼痛的标准治疗方法，已经显示出能有效减轻围手术期疼痛、改善肺功能、降低手术应激反应和衰减交感神经激活等优点。但是其操作比较复杂且具有较高的技术含量，容易发生神经的直接损伤、周围血肿、甚至压迫脊髓导致截瘫等严重并发症。

### 2.3 椎旁阻滞

当有硬膜外阻滞的禁忌症时，椎旁阻滞可替代硬膜外阻滞^[11]^。椎旁阻滞通过在背根水平阻断身体同侧的肋间神经传导而发挥其止痛效果。在Gulbahar^[13]^小组的试验中，50例接受后外侧开胸手术的患者，分成硬膜外阻滞和椎旁阻滞两组，评估了术后3天内每3 h的VAS评分、氧饱和度、心率、血压、肺活量测定（每12 h）、动脉血气分析。术后3天内，两组之间的各项评估指标均无明显差异，椎旁阻滞组在术后无并发症发生，而硬膜外阻滞组则有5例患者被检测到有并发症发生。椎旁阻滞主要优点是操作简单、单侧的神经阻滞、不易造成低血压、尿潴留、神经损伤和下肢功能减退等并发症。

### 2.4 肋间神经阻滞

通过阻滞肋间神经传导来减轻术后疼痛，但有时候它需要大剂量的局部麻醉，因此较易造成全身毒害^[12]^。

### 2.5 皮肤电神经刺激（transcuataneous electrical nerve stimulation, TENS）

TENS是通过皮肤将特定的低频脉冲电流输入人体以治疗疼痛的电疗方法。最早从上世纪七十年代早期开始，TENS即作为几种内科或手术相关的急、慢性疼痛的辅助治疗方法。最近Fiorellia^[11]^的一项研究将50例标准的后外侧开胸患者术后分成两组：研究组接受TENS治疗，安慰组进行相同操作，但是电极片中无电流输出。每组各有25例，评估术后6 h、12 h、24 h、48 h、72 h、96 h和120 h的血清细胞因子、疼痛积分、呼吸功能、镇痛泵镇痛药使用量，结果表明在各项评估因素中，TENS组表现出明显的优势，可见TENS作为开胸术后辅助镇痛方法，有一定价值。

## 3 总结

尽管已有诸多相关研究在手术方式和药物镇痛两个方面对治疗和预防开胸术后疼痛进行了一些探索，但由于疼痛的巨大个体差异，手术者操作技术的差别以及研究样本量的限制，目前尚难得出确切的结论支持其中的某种方法为“最优”选择。这一领域仍期待着临床专家的深入探索和研究。
